# Parent-Child Separations and Mental Health among First Nations and Métis Peoples in Canada: Links to Intergenerational Residential School Attendance

**DOI:** 10.3390/ijerph19116877

**Published:** 2022-06-04

**Authors:** Robyn J. McQuaid, Flint D. Schwartz, Cindy Blackstock, Kim Matheson, Hymie Anisman, Amy Bombay

**Affiliations:** 1 Department of Neuroscience, Carleton University, Ottawa, ON K1S 5B6, Canada; kimmatheson@cunet.carleton.ca (K.M.); hymie.anisman@carleton.ca (H.A.); 2 University of Ottawa Institute of Mental Health Research at the Royal, Ottawa, ON K1Z 7K4, Canada; 3 Department of Psychology and Neuroscience, Dalhousie University, Halifax, NS B3H 4R2, Canada; fschwartz@dal.ca; 4 First Nations Child and Family Caring Society, Ottawa, ON K1R 7S8, Canada; cblackst@fncaringsociety.com; 5 School of Social Work, McGill University, Montreal, QC H3A 1B9, Canada; 6 Department of Psychiatry and School of Nursing, Dalhousie University, Halifax, NS B3H 4R2, Canada; amy.bombay@dal.ca

**Keywords:** child welfare, depression, Métis, First Nations, parent-child separations, psychological distress, residential school

## Abstract

First Nations children are over 17 times more likely to be removed from their families and placed in the child welfare system (CWS) than non-Indigenous children in Canada. The high rates of parent-child separation have been linked to discriminatory public services and the Indian Residential School (IRS) system, which instigated a multi-generational cycle of family disruption. However, limited empirical evidence exists linking the IRS to subsequent parent-child separations, the CWS, and mental health outcomes among First Nations, Inuit, and Métis populations in Canada. The current studies examine these relationships using a nationally representative sample of First Nations youth (ages 12–17 years) living in communities across Canada (Study 1), and among First Nations and Métis adults (ages 18+ years) in Canada (Study 2). Study 1 revealed that First Nations youth with a parent who attended IRS had increased odds of not living with either of their biological parents, and both IRS and not living with biological parents independently predicted greater psychological distress. Similarly, Study 2 revealed that First Nations and Métis adults with familial IRS history displayed greater odds of spending time in the CWS, and both IRS and CWS predicted elevated depressive symptoms. The increased distress and depressive symptoms associated with parent-child separations calls for First Nations-led interventions to address the inequities in the practices of removing Indigenous children and youth from their families.

## 1. Introduction

The 2019 cycle of the Canadian Incidence Study on Reported Child Abuse and Neglect found that First Nations children were over 17 times more likely to be placed in out of home care than non-Indigenous children, which was driven by poor housing, poverty, substance use and domestic violence [1]. Statistics Canada data also show a disproportionate number of First Nations, Métis and Inuit children do not live with at least one of their biological (birth) parents, compared to the general Canadian population [2]. This is similarly the case in other colonized countries such as Australia and the United States [3,4]. The preponderance of literature and the Canadian Courts have linked the disproportionate separation of First Nations children from their families to structural risk factors arising from colonialization [1,5,6,7,8], and discriminatory child and family services that are inequitable and fail to account for the actual needs and cultural realities of First Nations children [9,10].

The intergenerational consequences of colonial policies and practices [8,11] have promoted ongoing inequities in relation to health and social resources for First Nations communities [12]. Such disparities have been linked to the cumulative effects of colonization, including systemic discrimination, displacement, and forced assimilation policies [7,8,9,10,13,14]. The Truth and Reconciliation Commission of Canada described the Indian Residential School (IRS) system as cultural genocide. The Canadian government forced generations of First Nations, Inuit, and Métis children from their families and communities to attend “schools,” that were more akin to re-education camps, operated by Christian churches with the aim of assimilation. Operating from the mid-1800s until the 1990s, children in the schools experienced institutional neglect, overcrowded conditions, malnutrition, the spread of preventable communicable disease, as well as widespread physical, psychological, and sexual abuse and death [7,8,15]. The IRS system, together with other harmful government policies and practices, severed cultural, community, and family ties, disrupted the continuity of parenting, and has been associated with multi-generational impacts on health and well-being [13,16,17,18,19].

It has been maintained that continuing high rates of First Nations parent-child separation are rooted in the IRS system, which resulted in a cycle of parental disruption [12,20,21]. However, few studies have empirically explored the link between IRS exposure, subsequent parent-child separations, and distress levels. Existing studies typically comprised non-representative samples of Indigenous adults and/or young people living off-reserve [11,22,23,24]. The current studies explored the link between intergenerational IRS exposure (i.e., those who had a parent and/or grandparent who attended IRS), parent-child separation, and youth psychological distress in a nationally representative sample of First Nations youth living on-reserve and in northern communities in Canada (Study 1). This work was then extended to examine child welfare system (CWS) exposure, linking CWS experiences to IRS family history and depressive symptoms among a non-representative sample of Indigenous adults in Canada (Study 2).

### 1.1. The Indian Residential School System

The IRS system, which operated from the 1880s until 1996, was established by the government of Canada for the purpose of assimilating Indigenous children [7,8,13,25]. Multiple generations of Indigenous children were separated from their parents, and forced to live in institutions where they endured the denigration of their cultural values, severe forms of physical and sexual abuse and neglect, and high rates of disease and mortality [7,8,26]. While some children overcame the trauma, overall survivors have presented with higher rates of negative health outcomes compared to those who had not attended an IRS [19,27]. Multi-generational effects of IRSs have also been documented in survivors’ children and grandchildren. In this regard, First Nations adults and youth living on- and off-reserve with parents and/or grandparents who attended IRS, reported higher levels of psychological distress, suicidal thoughts and attempts, substance use, and poorer self-reported mental and physical health compared to their First Nations counterparts without a family history of IRS attendance [17,28,29,30,31,32].

Multiple pathways exist through which the children and grandchildren of IRS survivors came to be at elevated risk for negative health outcomes. Aside from the greater childhood and lifetime trauma associated with familial IRS attendance, negative outcomes have been linked to discriminatory public services [10], and socioeconomic, household, and community-level risk factors in subsequent generations [11,18,27,33]. Furthermore, IRS survivors were removed from their parental role models and traditional family practices at a young age. Thus, it is understandable that some survivors might have experienced challenges in parenting their children [34,35,36].

### 1.2. The Overrepresentation of First Nations Children in the Child Welfare System in Canada

First Nations children are over 17 times more likely to be placed in child welfare care than non-Indigenous children [1]. While there is a lack of national data on placement rates for Métis and Inuit children, Statistics Canada (2016) [37] noted that Indigenous children represent 7.7% of the Canadian population and Indigenous children represent over half (52.2%) of children age 14 and younger in foster care. There is growing evidence that the overrepresentation of Indigenous children within the CWS can be ascribed to discriminatory public services [9,10] and social and economic inequities [1,11,22], which were linked to governmental forced assimilation policies. Prospective cohort studies indicated that Indigenous youth in British Columbia whose parents attended IRS were at elevated risk of having been in the CWS [22,23]. Beyond the influence of the IRSs, the situation was exacerbated by the ‘Sixties Scoop’ [12,21], a period from 1950 to 1990, during which about 20,000 Indigenous children were removed from their families and communities by the CWS [21,38,39]. The Sixties Scoop represented a shift in the practice of assimilationist goals; as IRSs began to close, Indigenous children were instead placed with non-Indigenous foster parents, adoptive parents, and in group homes, often permanently separated from their families and community [39,40,41]. Indigenous adults born during the Sixties Scoop era whose parents attended IRS were at greater risk for experiencing cumulative childhood household adversity, thus increasing the likelihood of CWS placement [11]. The impact of such policies, however, continued long after the Sixties Scoop through systemic inequities.

More recently, a landmark human rights case found that Canadian government responsible for discriminating against First Nations children and families by under-funding First Nations child welfare services and failing to implement Jordan’s Principle to ensure non-discrimination in other public services [9]. This discriminatory conduct coupled with CWS laws that codify structural risks as parental deficits contribute to First Nations children being more likely to be removed from their families than during Residential Schools or the Sixties Scoop [5].

### 1.3. Health and Social Outcomes Associated with Parental-Child Separations

The concept of shared child care within a family and community, sometimes referred to as customary adoption, or customary care, is a common traditional practice among Indigenous communities [42,43,44]. In customary adoption, children know who their birth parents are and kinship ties are strengthened, which may promote resilience among children, families, and communities [42,44]. In this regard, a prospective study of Inuit kinship/customary adopted children and non-adopted children found that despite lower socioeconomic status in adoptive households, adoption status was not a risk factor for behavioral problems after controlling for prenatal factors [43]. It was suggested that customary adoption and care allows for a “layering of family” and promotes language, culture, and identity [44]. However, empirical exploration on these outcomes is preliminary.

In contrast to customary adoptions, qualitative reports indicated that Indigenous adoptees into non-family member households during the Sixties Scoop described experiencing loss of identity and culture, social exclusion, abuse, feelings of cultural shame and disconnection as well as substance use, mental health problems, homelessness, and incarceration [38,40,45,46]. In fact, Indigenous adoptees in the United States report greater mental health problems and higher odds of poverty and poly-victimization than White adoptees, although the study did not specify the ethnicity of the adoptive parents, limiting the conclusions that can be drawn from the results [47].

Still yet another context in which children are separated from their biological parents includes institutionalization and foster care. While there is limited national child welfare data in Canada owing to the lack of a national database [8], available studies link interactions with the CWS with negative health and social impacts among Indigenous youth and adults in Canada [23,48]. For instance, one study found CWS placement was strongly linked to distress among Indigenous youth [49]. Analyses using the Aboriginal Peoples Survey (wherein participants resided ‘off-reserve’) administered in 2006, revealed that Métis adults with a history of foster care placement were more likely to have had a recent depressive episode and lifetime suicide ideation than those who had never been in care, and this relationship was mediated by exposure to adverse childhood experiences [48]. In line with these findings, a prospective study with an at-risk population of urban Indigenous youth in British Columbia found that CWS placement (majority in foster care, but also group homes, kinship care, and adoption) was linked to self-harm, suicide ideation and attempts, drug overdose, mental illness, homelessness, and risky behaviors [23]. In contrast, among this same cohort of Indigenous youth living in British Columbia, growing up traditionally, currently following their traditional culture, knowing their Indigenous language, and having sought treatment was associated with greater resilience [50].

## 2. The Current Research

The current studies were conducted to examine associations between the intergenerational IRS system exposure and parent-child separations with mental health outcomes. First the links between intergenerational IRS exposure, parent-child separation, and youth psychological distress were explored in a nationally representative sample of First Nations youth living on-reserve in Canada (Study 1). This was then extended to evaluate the associations between CWS experiences, IRS family history, and depressive symptoms among Indigenous adults in Canada (Study 2).

### 2.1. Study 1: Representative Sample of First Nations Youth Living in Their Home Communities

Analyses of the youth-specific 2015/16 First Nations Regional Health Survey (RHS) assessed the relationships between intergenerational IRS exposure, living arrangements (i.e., living with at least one birth parent), and levels of psychological distress among First Nations youth living in communities across Canada. It was hypothesized that:

(1) Youth with a parent and/or grandparent who attended IRS would be at increased odds of not living with at least one biological parent.

(2) Youth who had a parent and/or grandparent who attended IRS would report greater levels of psychological distress compared to those whose parents/grandparents did not attend.

(3) Youth who do live with at least one biological parent would report lower levels of psychological distress compared to those who did not live with one or both parents.

#### 2.1.1. Methods

##### Participants and Statistical Procedures

Data from the 2015/16 youth First Nations Regional Health Survey (RHS) were analyzed. Participants comprised 4968 (weighted = 47,918) youth living in First Nations communities across Canada between the ages of 12 to 17 years (Source: FNIGC, “2018-MCQUR-001-2020 Apr 16-Youth”, 16 April 2020). The mean age of the sample was 14.85 years (95% CI [14.7, 14.9]); 50.6% self-identified as male and 49.4% as female. A stratified 2-stage sampling design was used which is based on the 2014 Indigenous and Northern Affairs Canada Registry count. The first sampling stage involved selecting specific communities, according to region, sub-region and community size. The second sampling stage involved selecting individuals within each community using band membership lists. The RHS is governed by the First Nations Information Governance Centre (FNIGC), whose Regional Member Organizations represents different regions, and follows the First Nations principals of OCAP^®^ (Ownership, Control, Access, and Possession). FNIGC approved this study and was given the opportunity to review the manuscript. However, the analyses and interpretation do not necessarily reflect the views of FNIGC and any statistics reproduced from these results must be accompanied by a citation of this article. This study was approved by research ethics boards at Dalhousie University (REB #2018-4585), the Royal Ottawa Institute of Mental Health Research (REB #2018-020) and Carleton University (REB #115083). The Complex Samples Module of SPSS version 20 ( IBM) was used for all analyses to produce estimates based on weights and specifications of the RHS complex sampling design. Participants with missing data on relevant variables for specific analysis were excluded from that analysis. Estimates with high coefficients of variation (greater than 33.3%) and/or small cell counts (fewer than 5 individuals) were suppressed, and values marked with ^E^ represent estimates with high coefficient of variation (between 16.6% and 33.3%), requiring cautious interpretation. Cross-tabulations were conducted between categorical variables, including familial IRS attendance and living with biological parents. A binary logistic regression assessed the association between familial IRS attendance and the odds of not living with at least one biological parent. Analyses of covariance (ANCOVA) controlling for age and gender assessed the associations between familial IRS attendance and living with biological parents and psychological distress.

#### 2.1.2. Measures

The youth survey included questions on age (continuous), and gender (male or female). There is a separate question in the RHS that asks youth aged 15–17 if they identified as Transgender and/or Two-Spirited. However, since the current study includes youth of all ages (12–17 years), we could only consider youth identifying as male or female. Youth were asked who they lived with, and based on their responses were categorized into three groups: (1) Does not live with either biological parent; (2) lives with one biological parent; (3) lives with both biological parents. IRS familial experiences were determined by asking participants whether they or their mother/father or any of their grandparents had attended IRS. Since we were interested in the intergenerational consequences of IRS, individuals who themselves had attended IRS were excluded from all analyses. Four groups were then created including: (1) those reporting that no parent or grandparent attend (no IRS experience); (2) those who had a grandparent attend; (3) those who had a parent attend; and (4) those who had both a grandparent and parent attend.

Psychological distress was measured using the 10-item Kessler scale (K10) [51]. This scale assesses symptoms of anxiety and depression in the past month, with each item ranging from 1 (none of the time) to 5 (all of the time). Total scores are summed and can range from 10 to 50, with higher scores indicating higher distress. The K10 total score was validated as a measure of distress with strong reliability among First Nations populations [31,52].

#### 2.1.3. Results

##### Descriptive Analyses

Descriptive statistics on living arrangements, IRS family history, and distress scores are shown in Table 1.

##### Familial IRS Attendance and Living with a Biological Parent

**Figure 1:**
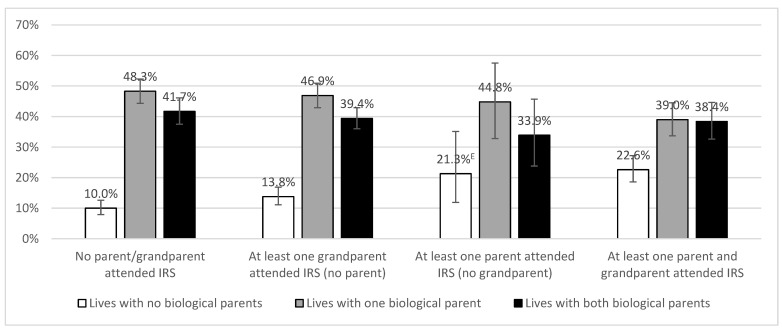
Proportion of youth who did not live with any biological parents, or live with one or two biological parents according to familial IRS history (Source: FNIGC, “2018-MCQUR-001-2020 Apr 16-Youth”, 16 April 2020). ^E^ = interpret with caution.

As shown in Figure 1, the proportion of youth living with no biological parents was higher with increasing IRS history. An opposite pattern was displayed among youth living with one or two biological parents. Thus, for subsequent logistic regression analyses the latter two groups were combined and compared to youth who lived with no biological parents. Moreover, given that the frequency of individuals living with no biological parents was similarly elevated among individuals who had a parent (but no grandparents) who attended IRS and those who had both a parent and grandparent who attended; these categories were thus combined in the subsequent logistic regression. The resulting three category IRS variable compared youth whose parents and grandparents did not attend, those with at least one grandparent (but no parent) who attended, and those who had at least one parent who attended (regardless of whether or not a grandparent also attended or not).

As shown in Table 2, logistic regression analysis including age and gender in the model revealed that the odds of not living with either biological parent was higher among youth with a parent who attended IRS compared to those with no parents or grandparents who attended IRS. Increasing age was associated with an increased likelihood of not living with either biological parent, whereas gender was not related to living with biological parents. Moreover, in a separate model, gender did not interact with familial IRS attendance, *p* = 0.99.

##### Familial IRS Attendance & Not Living with Biological Parents Predicting Distress

Youth who reported that none of their parents or grandparents attended IRS reported significantly lower levels of distress (*M* = 16.67), 95% CI [16.09, 17.26], compared to those with a grandparent who attended (*M* = 18.90), 95% CI [18.28, 19.52], those with a parent but no grandparent who attended (*M* = 22.39), 95% CI [19.20, 25.59] and those with a parent and grandparent who attended (*M* = 21.40), 95% CI [20.11, 22.68] (Source: FNIGC, “2018-MCQUR-001-2020 Apr 16-Youth”, 16 April 2020). Since distress levels were similar between those with a parent who attended IRS (but no grandparent) and those who had both a parent and grandparent who attended, they were once again combined into a single group in the subsequent ANCOVA.

Distress levels were higher among youth who did not live with any biological parent (*M* = 22.04; 95% CI [20.89, 23.20]) compared to those who lived with one biological parent (*M* = 18.54; 95% CI [17.87, 19.21]) and those who lived with both biological parents (*M* = 18.09; 95% CI [17.39, 18.78]) (Source: FNIGC, “2018-MCQUR-001-2020 Apr 16-Youth”, 16 April 2020). Based on these findings, a dichotomous variable that combined youths into those who did not live with at least one biological parent and those who lived with at least one birth parent was used in the subsequent ANCOVA analysis.

When examining IRS and living with birth parents in an ANCOVA with age and gender predicting distress levels, the model accounted for 11.4% of the variance in psychological distress. This model accounted for more variance than separate models considering IRS (10.1%) or living with biological parents (9.6%) alone. Familial IRS was significantly associated with distress, *F*(2, 142) = 32.93, *p* < 0.0001. Specifically, youth with a parent who attended IRS and youth with a grandparent (but no parent) who attended IRS had significantly elevated distress scores compared to youth with no family history of IRS, *p* < 0.0001 and *p* < 0.0001, respectively. Not living with a biological parent was significantly associated with greater distress compared to youth living with at least one biological parent, *F*(1, 143) = 13.97, *p* < 0.0001. Females reported greater distress than males, *F*(1, 143) = 107.64, *p* < 0.0001 and age was positively associated with distress, *F*(1, 143) = 20.96, *p* < 0.0001 (Source: FNIGC, “2018-MCQUR-001-2020 May 1-Youth”, 1 May 2020). Although familial IRS attendance and not living with any biological parent were associated with each other in the bivariate analyses and account for some shared variance in distress levels, in this analysis both remained significant and accounted for unique variance in distress. The interaction between parental IRS attendance and not living with biological parents in predicting distress was added to the model in a separate analysis but was not significant (*p* = 0.88).

#### 2.1.4. Summary

Analyses of population data revealed that IRS familial history was associated with subsequent parent-child separations. Specifically, First Nations youth living on reserves and in northern communities across Canada with at least one parent who attended IRS are less likely to live with a biological parent compared to youth without a parent or grandparent who attended IRS. Moreover, both parental and grandparent IRS history coupled with not living with a biological parent were associated with higher psychological distress scores, contributing some shared and unique variance. These data highlight that the cycle of removing First Nations children from their families continues today and can be linked back to the IRS system. In fact, First Nations children involved in child welfare investigations are more likely than non-Indigenous children to have a caregiver with a history of child welfare placement [1], further highlighting these harmful cycles. In Study 1 we were not able to specifically consider the child welfare system (CWS), thus, in Study 2, we examined the link between IRS and the CWS, as well as the associations to mental health outcomes.

### 2.2. Study 2: Sample of First Nations Adults Living Outside of Home Community

This study assessed the long-term effects of the Indian Residential School system on the distress levels of adult offspring. The goal of this study was to extend the findings from Study 1 by considering experiences with the CWS, linking these to IRS family history and distress levels among First Nations and Métis adults in Canada. Specifically, it was hypothesized that:

(1) Adults who had a parent and/or grandparent who attended IRS would be associated with increased odds of being exposed to the CWS.

(2) Adults who had a parent and/or grandparent who attended IRS would report higher depressive symptoms compared to those whose parents/grandparents did not attend.

(3) Adults who were exposed to the CWS would report greater depressive symptoms compared to those who were never apprehended.

#### 2.2.1. Methods

##### Participants and Procedures

Advertisements posted at Indigenous health and community centers located off reserve across Canada and electronic mailing lists related to Indigenous issues invited Indigenous adults (18 years and over) to participate in a study examining the long-term effects of IRS. Participants could complete the survey online or to have the questionnaires mailed to them. Participants provided informed consent, and after completion of the questionnaires, received a written debriefing and a gift certificate ($10). Since the focus of this study was on exploring the intergenerational impacts of the IRS system, individuals who reported that they personally attended IRS were excluded from the analyses. This study was approved by the research ethics board at Carleton University (REB #11-006).

The final sample comprised 433 adults (24.2% male; 75.5% female; one participant did not identify their gender), of which 17 completed the questionnaires by mail and 416 online. Individuals identified as Status First Nation (52.2%), non-Status First Nation (23.6%), and Métis (24.2%). The mean age of participants was 34.29 years (*SE* = 0.48; *Range* = 41). Participants lived across Canada with the greatest number of participants residing in Ontario (46.4%), followed by British Columbia (13.2%), Saskatchewan (9.5%), Alberta (9.2%), Manitoba and Quebec (8.1% each), 3.7% from the Atlantic provinces (New Brunswick, Nova Scotia, Newfoundland), and 1.2% from the Territories (Northwest Territory, Yukon, Nunavut), with n = 3 participants (0.7%) who did not report where they lived. This sample was previously used to examine the link between IRS, cumulative household adversity and the CWS but did not examine the links between the CWS and mental health outcomes [11].

#### 2.2.2. Measures

*Parent and/or grandparent attendance at Indian Residential School*. Participants were asked about their personal and family history of IRS, specifically if their parents and/or grandparents had ever attended. Of the included respondents who did not attend IRS personally, as in Study 1 there were four categories: (1) no parent and/or grandparent attended (no IRS experience); (2) at least one grandparent attended (but no parent attended); (3) at least one parent attended (but no grandparent attended); and (4) at least one parent *and* at least one grandparent attended.

*Involvement in the Child Welfare System*. Participants were asked whether they “ever spent time in the care of foster parents or in a group home,” and a separate question asked about who their primary caregiver(s) were while growing up, (multiple answers were allowed) with adoptive mother and adoptive father being listed as options. Researchers categorized participants as personally exposed to the CWS (no vs. yes) if they listed an adoptive parent as a primary caregiver and/or if they indicated that they had spent time in foster care or a group home (no vs. yes).

*Depressive Symptoms.* The 13-item Beck Depression Inventory short form (BDI-SF) [53] was used to assess depressive symptoms. For each item participants responded to one of four options ranging from low to high depression symptomatology. Total scores were summed across all items (α = 0.91), and ranged from 0 to 29.00 (*M* = 6.59, *SE* = 0.31)).

Statistical analyses conducted in Study 2, paralleled those conducted in Study 1. Namely, cross-tabulations were performed between categorical variables including familial IRS attendance and CWS experience. A binary logistic regression assessed the association between familial IRS attendance in predicting the odds of spending time in the CWS. ANCOVAs controlling for age and gender assessed the associations between familial IRS attendance and CWS experience in predicting depressive symptoms.

#### 2.2.3. Results

##### Descriptive and Bivariate Analyses

Almost twenty percent (19.4%) of participant had CWS experience. Participants differed in their IRS family history, with 40.2% reporting that none of their parents or grandparents attended IRS, whereas, 23.8% reported that their grandparent had attended, 18.0% had a parent who attended and 18.0% had both a grandparent and parent who attended.

##### Familial IRS Attendance and the Link to the CWS

**Figure 2:**
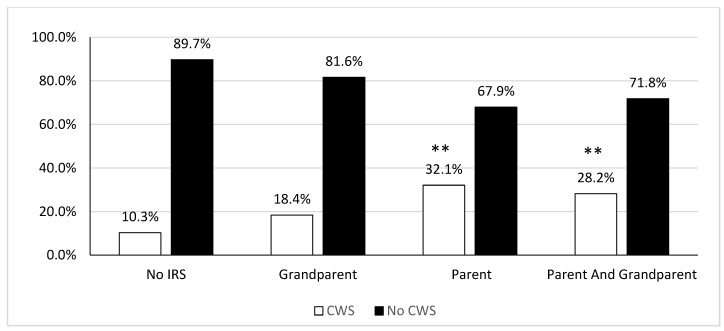
Frequency of individuals who spent time in the Child Welfare System by Residential School family history. ** *p* < 0.001, compared to no IRS family history.

As shown in Figure 2, time spent in CWS increased according to IRS familial experience. Specifically, individuals with a grandparent who attended IRS, but not a parent, spent more time in the CWS compared to those with no IRS family history, but this only approached significance, *χ*^2^ (1) = 3.65, *p* = 0.06. Individuals with a parent, but not a grandparent who attended IRS, as well as those with a parent *and* grandparent who attended, were both significantly more likely to spent time in the CWS compared those who reported no parent or grandparent attended IRS, *χ*^2^ (1) = 17.93, *p* < 0.001, and *χ*^2^ (1) = 12.87, *p* < 0.001, respectively. There were no significant differences in CWS experience between individuals with a parent who attended IRS only and those with both a parent *and* grandparent who attended IRS, *χ*^2^ (1) = 0.27, *p* = 0.60. Given that individuals with a parent, but not grandparent, who attended IRS did not differ from those who had both a parent *and* grandparent, in relation to CWS, they were combined into a single group (i.e., those with a parent who attended IRS) and compared to those who were not intergenerationally affected and those with a grandparent who attended for logistic regression analysis. Age and gender were included in multivariate analyses (results did not differ if age and gender were excluded from multivariate analyses).

The logistic regression model was significant, *χ*^2^ (4) = 34.58, *p* < 0.01, *R*^2^ = 0.12. As shown in Table 3, increasing odds of spending time in the CWS was found among individuals with a parent who attended IRS compared to adults with no family history of IRS. Moreover, those with a grandparent who attended IRS were also at increased odds of spending time in the CWS compared to individuals with no IRS family history. An older age was associated with increased odds of having spent time in the CWS. However, gender was not significantly associated with the likelihood of spending time in the CWS.

##### Familial IRS Attendance and CWS Predicting Depressive Symptoms

Depression scores differed according to IRS family history, *F*(3, 412) = 7.00, *p* < 0.001. Specifically, those with a grandparent who attended an IRS (*M* = 6.2 ± 0.61) did not significantly differ from individuals with no IRS familial history (*M* = 5.3 ± 0.47), *p* = 1.00; however, individuals with a parent (*M* = 7.9 ± 0.71) as well as individuals with both a parent and grandparent (*M* = 8.9 ± 0.74) who attended IRS, had higher depression scores compared to those without a parent or grandparent who attended family history of IRS, *p* = 0.02, and *p* < 0.001, respectively. Moreover, those with a parent who attended IRS and those with both a parent and grandparent who attended did not differ from each other in depression scores, *p* = 1.00. As there were no significant differences in depression scores between individuals with a parent, but not grandparent, and those who had both a parent *and* grandparent attend IRS, they were combined for the ANCOVA analyses. Depression scores were higher among individuals who spent time in the CWS (*M* = 9.00; *SE* = 0.79) compared to those who did not spent time in the CWS (*M* = 6.03; *SE* = 0.32), *t*(414) = −3.87, *p* = 0.001.

When IRS and living with birth parents were included in the same ANCOVA analysis with age, gender predicting distress levels, both spending time in the CWS *F*(1, 410) = 9.67, *p* = 0.002, *η*^2^ = 0.02, and parental IRS attendance *F*(1, 410) = 7.36, *p* < 0.001, *η*^2^ = 0.04, were significant when included in the same model. Specifically, individuals with a parent who attended IRS had higher depressive symptoms than those with a grandparent who attended, *p* = 0.04, and compared to those with no familial history of IRS, *p* < 0.001. Age, *F*(1, 410) = 0.74, *p* = 0.39, and gender, *F* (1, 410) = 0.22, *p* = 0.64, were not associated with depressive symptoms. In this analysis both IRS and CWS remained significant and accounted for unique variance in depressive symptoms, although it was interesting that the variance accounted for by IRS (5%) was diminished slightly (4%) when CWS was included in the model. The interaction between parental IRS attendance and time spent in the CWS in predicting depressive symptoms was added to the model in a separate analysis, but was not significant, *p* = 0.67. In this model, IRS and CWS remained significant predictors of depression scores, *p* = 0.002 and *p* = 0.008, respectively, whereas age and gender were not significant, *p* = 0.43, *p* = 0.59, respectively.

## 3. Discussion

Analyses of the RHS data in Study 1 revealed disproportionately high rates of parent-child separation in this nationally representative sample of First Nations youth living on-reserve and in northern communities. This is in-line with the 2019 cycle of the Canadian Incidence Study on Reported Child Abuse and Neglect that found that First Nations children were over 17 times more likely to be placed in out of home care than non-Indigenous counterparts [1]. Moreover, parent-child separations were strongly linked to IRS familial history, such that while 10% of First Nations youth who did not have any IRS family history reported not living with any biological parents, this was over 20% for First Nations youth who had a parent who attended IRS. This finding linking intergenerational experiences of IRS attendance to parent-child separations was mirrored in both the First Nations youth data (Study 1) and the Indigenous adult data (Study 2). Specifically, we found that First Nations youth and Indigenous adults who reported having at least one parent that attended IRS were at significantly increased odds of being separated from their biological parents and/or spending time in the CWS compared to those without an IRS family history. These findings are consistent with previous prospective studies of Indigenous youth in British Columbia that showed that having at least one parent who attended IRS was associated with twice the odds of having been in the care of the CWS compared to youth without familial IRS exposure [22,23]. In this same BC cohort, young Indigenous mothers who reported recent child removal, were more likely to have a parent who attended IRS, and most (70%) had been in CWS care themselves as children [24]. Moreover, among Indigenous adults from across Canada who were born during the Sixties Scoop era we found that parental IRS attendance increased the odds of spending time in the CWS, and that this relation was mediated by adverse childhood household experiences [11]. Together these results provide further evidence that disproportionately high rates of parent-child separation experienced by Indigenous populations should be considered in the context of colonial interventions that promoted and maintained child removal practices over multiple generations [1,7,12,13].

The current investigation contributed to the growing literature documenting associations between parent IRS attendance and the well-being of ensuing generations. Specifically, it was found that parent IRS attendance was associated with significantly higher distress in First Nations youth (Study 1) and depressive symptoms among Indigenous adults (Study 2) living in Canada. Although there is robust evidence demonstrating that IRS attendance negatively relates to the mental and physical health of IRS survivors and their children and grandchildren [19], few studies have carried out analyses of the RHS to assess how IRS is associated with well-being among First Nations youth living on-reserve (and none have done so in the most recent 2015/16 RHS data). Indeed, our findings are consistent with analyses of the 2008/10 youth RHS, in which parental IRS attendance was associated with elevated suicidal thoughts and attempts [28]. Similarly, Study 2 reveals higher depressive scores among First Nations and Métis adults with parental IRS attendances, which is consistent with our earlier analyses of the 2008/10 adult RHS data, in which we documented intergenerational and cumulative relations between parental and grandparental IRS experiences and suicide ideation and attempts in adult First Nations adults living in communities across Canada [17].

One of the overarching goals of this investigation was to examine the link between familial disruption through parent-child separation and spending time in the CWS with mental health outcomes. While few quantitative studies assessed this link among Indigenous populations in Canada, a national sample of Métis adults showed that children separated from parents through foster care predicted depression and suicide ideation in adulthood [48]. In line with this finding, Study 1 revealed that First Nations youth who did not live with at least one biological parent displayed significantly elevated psychological distress levels, and that this continued to be significant when familial IRS attendance was included in the same model. Similarly, in Study 2, Indigenous adults who spent time in the CWS showed significantly higher depressive symptoms, which also remained significant when familial IRS attendance was included in the model. Together these data indicate that while familial IRS and not living with a biological parent in subsequent generations are linked, they each account for unique variance in mental health outcomes. The IRS, Sixties Scoop, and present-day child welfare systems have been described as state-sponsored mechanisms of child removal that perpetuate the intergenerational transmission of trauma and institutionalization of Indigenous peoples [1,8,9,54]. By providing evidence of a link between institutionalization through the IRS system we can better understand the intergenerational impact of the IRS system: parental disruption and generational discontinuity.

The current findings add to the limited quantitative evidence assessing the links between familial IRS attendance, parental separations and the CWS, and the associated mental health outcomes. Importantly, these findings were obtained through nationally representative data of First Nations youth living on-reserve and in northern communities, and are consistent with the findings in a non-representative sample of First Nations and Métis adults. However, there are several limitations that ought to be considered. In Study 1, we were limited by the questions in the RHS. Specifically, a general measure of whether or not the child lived with at least one biological parent was limiting as the specific reasons for parent-child separations are unknown, and there were no questions about CWS involvement. It is possible that there may be significant differences in mental health outcomes between youth who were separated for various reasons. Another important limitation of Study 1 is that the RHS is focused on youth who are currently living on-reserve and in northern First Nations communities, and as a result the current analyses did not include data on First Nations youth who do not live in their home community for a variety of reasons, one of which could include institutionalized care. Moreover, 23% of youth had missing information to create the derived familial IRS history variable, and as such, they were not included in analyses comprising this variable. Study 2 also has a number of limitations including that it is a non-representative self-selected sample. However, pairing these data with nationally representative population-based data from Study 1, provides additional confidence in these findings. A benefit of Study 2, was that it extended the assessment of parent-child separations to more directly examine CWS involvement, although this question too was not without limitations. For example, the term ‘foster care’ is an all-inclusive term that could refer to in-home placements, group homes, and possibly kinship care. Thus, participants were considered to have been personally exposed to the CWS if they listed an adoptive parent as a primary caregiver and/or if they ever spent time in foster care or a group home, even though these experiences could have been vastly different. Moreover, while participants in Study 2 could have the survey mailed to them, on-line survey access could have been limited to First Nations peoples living on reserves as only 35% of First Nations households on reserve have broadband internet access [55].

In both studies, we were not able to run mediation analyses due to having a binary mediator. However, we were able to look at the unique variance owing to these variables separately and when both were included in the model. Moreover, distinct Indigenous groups and First Nations in different parts of the country differ with respect to traditions, histories, beliefs, and realities were analyzed together, and it would be valuable to explore regional differences. Indeed, while there are important variations across these groups, First Nations peoples (Study 1) and First Nations and Métis peoples (Study 2) have shared histories, including the IRS system, the Sixties Scoop. However important differences exist that are not accounted for in the current study. Finally, individuals who reported that none of their parents or grandparents attended IRS were categorized as ‘No IRS’. However, it is possible that they had other family members, (e.g., aunts/uncles) who attended. In fact, when considering IRS history, both studies rely on self-reporting of IRS personal and family history, and the TRC (2015) has documented the reluctance of some survivors to talk about their IRS experience so the sample may underestimate the number with a parent or grandparent with IRS experience.

## 4. Conclusions

Study 1 revealed disproportionately high rates of First Nations youth and adults are not living with their biological parents and are spending time in the CWS. It has been shown previously that the main contributing factor for First Nations children to be placed in CWS was neglect, a term used to remove children from their homes due to poverty [56,57]. While we did not evaluate the mediators or contributing factors underlying the parent-child separations in the current study, we previously showed that the relation between parental IRS attendance and CWS was mediated by a cumulative childhood household risk score [11]. In fact, the direct and intergenerational effects of IRS experiences, including poverty, mental health, and substance use issues are known risk factors for child neglect, removal and placement into foster care [39,58]. The current study quantitatively links the IRS to subsequent parent-child separations in the next generation, thus providing evidence emphasizing the need for interventions that address the structural discrimination contributing to poverty and culturally based interventions for those directly and intergenerationally affected by the IRS system. Moreover, the increased distress and depression scores in those who do not live with biological parents and spend time in the CWS, calls for culturally-relevant and First Nations-led interventions to address the structural inequalities that drive the over-representation of First Nations children in care that account for the multi-generational impacts of Residential Schools [5]. Additionally, kinship adoption or customary care practices may be protective by maintaining family and community connection [43,59,60], which could serve as a community alternative to foster care [42]. In fact, it is much more common for First Nations grandparents to take a central part in raising their grandchildren compared to the general population [61]. Thus, First Nations grandparents have historically played, and despite colonialism, continue to play significant roles in their grandchildren’s lives, which may foster resilience and well-being [62]. Together, the current studies suggest that systemic changes are required in the CWS to break the cycle of parent-child separations and promote healthy families that have equitable opportunity to live the lives they wish to have.

## Figures and Tables

**Table 1:** Characteristics of First Nations youth living on reserve and in northern communities across Canada (Source: FNIGC, “2018-MCQUR-001-2020 Apr 16-Youth”, 16 April 2020).

Characteristic	Weighted Proportion	95% Confidence Interval
Living Arrangement ^1^ (%)		
No biological parents	15.1	13.2, 17.2
One biological parent	45.3	42.6, 48.1
Both biological parents	39.5	37.0, 42.2
IRS Experience ^2^ (%)		
No IRS	33.7	31.0, 36.5
Grandparent	47.7	44.8, 50.7
Parent	2.3 ^E^	1.6–3.2
Parent and Grandparent	16.3	13.7–19.3
Psychological Distress ^3^ (mean)	18.9	18.4, 19.3
Male distress (mean)	16.7	16.3, 17.1
Female distress (mean)	21.1	20.4, 21.8

^1^ There were 149 participants (3.8% of full sample) who did not answer questions used to derive at least one of the variables in the analysis and were not included in the analysis (*n* = 4819 unweighted) ^2^ There were 1156 participants who did not answer questions used to derive at least one of the variables and were not included in the analysis (*n* = 3812 unweighted). ^3^ There were 457 participants who did not answer this question and were not included in analysis on distress (*n* = 4511 unweighted). ^E^ = interpret with caution.

**Table 2:** Logistic regression analysis predicting not living with either biological parent (Source: FNIGC, “2018-MCQUR-001-2022 Mar 24-Youth”, 24 March 2022).

Model	β	SE	Significance	OR (95% CI)
IRS Experience (ref = no IRS history)				
Parental Attendance	0.95	0.17	<0.001	2.57 (1.84–3.59)
Grandparent Attendance	0.60	0.16	=0.07	1.41 (0.98–2.03)
Age	0.10	0.03	0.007	1.10 (1.03–1.18)
Gender (ref = male)	0.04	0.15	0.77	0.96 (0.71–1.29)

**Table 3:** Logistic regression analysis predicting time spent in the Child Welfare System.

Model	β	SE	Significance	OR (95% CI)
IRS Experience				
Parental Attendance	1.18	0.31	<0.001	3.26 (1.78–5.99)
Grandparent Attendance	0.81	0.36	0.03	2.25 (1.10–4.60)
Age	0.05	0.01	<0.001	1.05 (1.02–1.08)
Gender	−0.10	0.29	0.73	0.91 (0.51–1.60)

## Data Availability

The RHS data used for Study #1 is governed by the First Nations Information Governance Centre (FNIGC), and access to these data are made through a data request and application process. For Study #2, these data were not approved by the REB to be made publicly available.
